# Validity of Willingness to Pay Measures under Preference Uncertainty

**DOI:** 10.1371/journal.pone.0154078

**Published:** 2016-04-20

**Authors:** Carola Braun, Katrin Rehdanz, Ulrich Schmidt

**Affiliations:** 1 Kiel Institute for the World Economy, Kiel, Germany; 2 Department of Economics, University of Kiel, Kiel, Germany; 3 Department of Economics and Econometrics, Universiy of Johannesburg, Johannesburg, South Africa; University of Reading, UNITED KINGDOM

## Abstract

Recent studies in the marketing literature developed a new method for eliciting willingness to pay (WTP) with an open-ended elicitation format: the Range-WTP method. In contrast to the traditional approach of eliciting WTP as a single value (Point-WTP), Range-WTP explicitly allows for preference uncertainty in responses. The aim of this paper is to apply Range-WTP to the domain of contingent valuation and to test for its theoretical validity and robustness in comparison to the Point-WTP. Using data from two novel large-scale surveys on the perception of solar radiation management (SRM), a little-known technique for counteracting climate change, we compare the performance of both methods in the field. In addition to the theoretical validity (i.e. the degree to which WTP values are consistent with theoretical expectations), we analyse the test-retest reliability and stability of our results over time. Our evidence suggests that the Range-WTP method clearly outperforms the Point-WTP method.

## Introduction

There is a long-lasting and ongoing debate about the best method to elicit willingness to pay (WTP) for contingent valuation (CV) of non-market goods in environmental [1], agricultural [2, 3] and health economics [4, 5]. A fundamental issue in this debate is the question whether open- or closed-ended methods lead to more reliable results [6–8]. Traditional CV studies rely on an open-ended approach in which respondents are free to state any amount they wish to indicate their WTP [9, 10]. The open-ended method has been criticized for the high cognitive load it imposes on respondents, a high number of observed non- or zero responses, and the fact that the distribution of responses is typically heavily skewed towards high amounts which might be caused by a strategic bias [11]. As an alternative, closed-ended methods have been developed where respondents select a value from a pre-specified list (payment card) or where WTP is derived from (a series of) dichotomous choices. Despite the increasing popularity of closed-ended methods, several methodological issues have been observed. While approaches relying on payment cards are subject to starting point and range biases (see e.g. [12–14]), dichotomous choices are statistically inefficient and may be prone to the ‘yea-saying bias‘, i.e. a tendency to accept presented amounts [14–19]. In view of these issues a number of authors have questioned the superiority of closed-ended over open-ended methods [7, 8, 14, 17, 20–22].

A particular factor which contributes to noisy or even biased measurements of WTP is preference uncertainty. Preference uncertainty may be caused by incomplete knowledge about the features of the object under evaluation or simply by the fact that a person is unsure about her own preferences [23–27]. Since individuals are typically unable to gain experience of non-market goods through repeated purchase and consumption, preference uncertainty is highly relevant for CV studies [28]. Due to vagueness of preference, people often have only regions of indifference instead of well-defined indifference curves [29–32], a fact that theoretically contradicts the existence of a single Point-WTP [33–35].

Several CV studies based on closed-ended surveys take preference uncertainty into account. Ready et al. [36], for example, allow for six response categories in their survey ranging from ‘definitely yes‘ to ‘definitely no‘. Welsh and Poe [37] apply a similar approach. Li and Mattsson [38] and Champ et al. [39] allow respondents to express their uncertainty on scales from 0% to 100% or from 1 to 10. However, all these approaches involve several problems. All respondents must interpret the employed scales in the same way and be able to assess their degree of preference uncertainty correctly [40, 41]. Moreover, it is not at all clear how to interpret the obtained information. For instance Hakansson [41] argues that the statement to be 60% certain to pay US$100 for a given project should not necessarily be treated as an average WTP of US$60. As response to this critique, several studies have addressed preference uncertainty by using payment cards to elicit lower (the maximum amount the individual is sure to be willing to pay) and upper (the minimum amount above the individual is sure to refuse to pay) bounds of WTP [28, 34, 42–46]. The length of the interval between lower and upper bound can be regarded as a measure of preference uncertainty in these studies. However, the above mentioned general problems of closed-ended approaches, in particular the starting point and range biases remain.

The only open-ended CV study which addresses preference uncertainty has been performed by Hakansson [41]. The design of Hakansson allows respondents to state either an exact amount or an interval of two amounts as their WTP. The interval is elicited by responses to the statement ‘I am willing to pay between … and …’. This means that the bounds of the interval are not clearly defined as lower (upper) bound of WTP, i.e. the maximum (minimum) amount for which the individual is sure to (refuses to) pay as in the closed-ended studies mentioned above. Consequently, respondents could differ in the interpretation of the interval bounds and also in the degree of uncertainty at which they switch from stating an exact amount to giving an interval. Nevertheless, the work of Hakansson [41] led to rather promising results.

Recently, a new open-ended WTP elicitation approach was introduced in the marketing literature [35, 33]. Therein, WTP is elicited as a range with properly defined bounds as in the previous closed-ended studies. We call this approach Range-WTP in comparison to the traditional open-ended Point-WTP where only a single value is elicited. Until now, this method has been only used for the valuation of market goods, i.e. chocolate and wine.

Our paper has three main goals: Our first goal is to apply the Range-WTP to CV studies for the valuation of non-market goods. While Wang et al. [35] show that the Range-WTP outperforms the Point-WTP for predicting purchase probabilities of consumer goods, it is still unclear whether this holds also for our study since preference uncertainty is supposedly much larger for non-market goods. Our second goal is to compare the performance of Range- and Point- WTP for CV studies. In doing so, we use the criterion of theoretical validity of Range-WTP and compare it to that of Point-WTP. Therefore, we measure the degree to which WTP values are consistent with theoretical expectations. More precisely, we measure other subjective evaluations of our non-market good and test whether they are related to WTP measures in a consistent way (see Section 3.3 for details). We also check for the stability of our results over time. In addition, we analyse the test-retest reliability by considering the correlation between the same respondents’ answers at two different points in time. In a broader sense we want to contribute to the issue whether the earlier criticism on open-ended methods can be watered down by eliciting ranges. To the best of our knowledge, no previous paper has compared theoretical validity of two open-ended methods. Our third goal is to provide empirical evidence for our test case, the avoidance of the use of solar radiation management (SRM), a climate engineering technique that aims to counteract climate change by the injection of sulphate aerosols into the stratosphere [47, 48]. Apart from mitigation of carbon emissions, SRM has been discussed as an option for achieving the two-degree climate target. Since most people have never heard of SRM (see e.g. [49, 50]), preference uncertainty should be rather high for SRM. This is ideal for testing the performance of Range-WTP.

To compare the theoretical validity of Range- and Point-WTP, we conducted two large online surveys on SRM in Germany with a total number of nearly 1,500 participants. Both surveys were identically structured and all respondents received the same information before stating their preferences. However, in Survey A we elicited Point-WTP whereas in Survey B we elicited Range-WTP. This provides for an adequate comparison of response differences. To test for the stability of our results over time and for the test-retest reliability of both methods, we conducted a follow-up study in which about 800 of the initial 1,500 respondents participated.

The remainder of the paper is organized as follows: Section 2 introduces our conceptual framework and provides an overview of the elicitation approach. Section 3 outlines our data and survey design (3.1), WTP elicitation in more detail (3.2) and the test of validity and reliability (3.3). Section 4 discusses the results and section 5 concludes.

## Conceptual Framework

**Fig 1:**
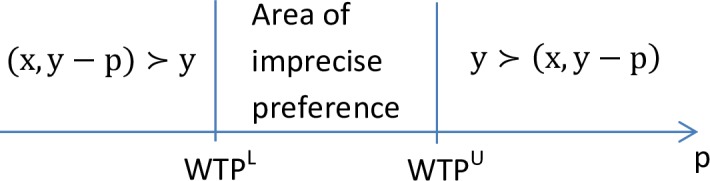
Imprecise preferences.

As argued above, asking for single Point-WTP may not be justified in the case of preference uncertainty, i.e. if well-defined indifference curves do not exist. To see this formally, consider a person with initial wealth y who is thinking about buying good x. Obviously, she will buy the good for price p if (x, y-p) ≻ y, where ≻ is the asymmetric part of her binary preference relation. In the case of thick indifference curves, the Point-WTP implicitly given by (x, y–WTP) ~y may not be well-defined. However, there exist an upper (WTP^U^) and lower (WTP^L^) bound of WTP defined by WTP^U^ = inf{p | y ≻ (x, y–p)} and WTP^L^ = sup{p | (x, y–p) ≻ y}, see Fig 1. If preferences are continuous, i.e. if indifference curves are thin, the area of imprecise preferences will collapse such that WTP^U^ = WTP^L^ = Point-WTP.

Wang et al. (2007) were the first to develop an open-ended approach where WTP is measured as the range between WTP^L^ and WTP^U^ rather than as a point. In a set-up involving 175 customers, they test the range approach in an experimental elicitation of consumers’ reservation prices for chocolate and red wine. They assume linear decreasing purchase probabilities between the lower bound (100%), indifference range (50%) and upper bound (0%). A lottery ensures incentive compatibility, e.g. if the randomly drawn lottery price is lower than the lower bound, then the respondent must buy the good at the drawn lottery price. They conclude that Range-WTP yields better predictions for actual buying probabilities than Point-WTP. Dost and Wilken [33] build on the Wang et al. [35] approach but drop the elicitation of an indifference point and restrict the assumption of linear decreasing purchase probabilities. They test the range on a study of *caffè latte* and show that the traditional Point-WTP approach reveals the midpoint of the range. The only non-market CV study is Hakansson [41]. Hakansson [41] elicits the WTP for increasing the number of wild salmon in a Swedish river. She proposes a similar approach though, as pointed out above, the interval bounds in her study are not clearly defined as WTP^L^ and WTP^U^.

Our approach redefines, applies and tests the Range-WTP method proposed by Wang et al. [35]. Building on their initial approach, we ask respondents to provide a lower-bound WTP and an upper-bound WTP. Logic suggests that the upper bound should be identical to or exceed the lower bound of WTP. Unlike Wang et al. [35], we apply the Range-WTP method to a public good rather than to consumer goods such as chocolate or red wine. We follow Dost and Wilken [33] and drop the elicitation of an indifference price. We do not incentivise respondents and abstain from use of a lottery. This ensures that the method is survey-compatible and easy to understand for survey respondents. While Wang et al. [35] show that Range-WTP is a better predictor of actual purchase probabilities for consumer goods as compared to Point-WTP, it remains an open question whether the range method is suitable for CV studies on non-market goods. This is particularly true as the marketing studies have applied the range method only to goods with very low preference uncertainty (chocolate, wine, coffee) while for our good (SRM) preference uncertainty should be rather high.

In order to investigate the suitability of Range-WTP for CV studies, theoretical validity, test-retest reliability and stability of results over time are important criteria. Theoretical validity, which is a subgroup of construct validity [51], can be defined as the degree to which the empirical findings are consistent with theoretical expectations [12]. A number of existing studies compare the theoretical validity of different evaluation measures for environmental goods (e.g. [51–53]). Closely related to our study are Bateman et al. [54, 55], who in a study on national parks in the UK compare the theoretical validity of Point-WTP with that of the iterative bidding approach. The authors find that in line with theoretical expectations, the values elicited by Point-WTP are determined by a number of socio-economic factors such as income. Accordingly, they conclude that Point-WTP fulfils the criterion of theoretical validity. By contrast, Ressurreicao et al. [56] find that in their study on species loss in the open sea Point-WTP does not fulfil the criterion of theoretical validity. The authors show that in terms of theoretical validity the closed-ended payment card method outperforms the Point-WTP method. Ressurreicao et al. [56] argue that the Point-WTP method may not be suitable for more complex issues, as they consider the valuation of all marine species a too complex a task for respondents. Unlike our paper, these and other existing studies compare the traditional open-ended Point-WTP method with a closed-ended method.

As far as we know, no study has yet compared the theoretical validity of two open-ended methods, as we do in this paper by comparing the open-ended Point-WTP method with the open-ended Range-WTP method. Nor are we aware of any studies that have analysed whether results concerning theoretical validity are stable over time. We also investigate the test-retest reliability of the Range- and Point-WTP methods. Test-retest reliability of WTP measures is defined as the correlation between responses measured at different points in time with the same sample [57] and has been addressed in several previous CV studies (e.g. [58, 59]).

## Methodology and Data

### Ethics statement

Our data comes from an internet survey organized by a professional survey institute. Participants are registered at this institute and receive a monetary compensation for every survey they perform. Our survey only involved questions about sociodemographic data and personal attitudes without e.g. the possibility of losing money or incurring any other harm. Authors have access to anonymized personal information of respondents (birth date, occupation, income class, but not to names, address). All data was provided by the respondents voluntarily, i.e. they did not have to answer these questions. All data was anonymized by the survey institute.

Ethical approval for such type of surveys is required neither by our academic institution nor by the survey institute. There was no waiver of ethical approvement obtained for this study. Participants registered for the survey and agreed to participate (written consent). They could leave and stop the survey at any time; the answer ‘don’t know’ was possible for every question.

### Data and Survey Design

Our study uses novel data from two surveys of representative internet users on SRM that we conducted in July 2014 and in August 2013 with two follow-up surveys in August 2014. The surveys were structured identically and differed only with respect to the WTP elicitation method. In *Survey A*, conducted in July 2014, WTP was elicited using the Point-WTP method. In *Survey B*, conducted in August 2013, WTP was elicited using the Range-WTP method. Our working samples consist of 776 (Survey A) and 663 (Survey B) observations. In the two follow-up studies conducted in August 2014 (referred to as Surveys A* and B*), we repeated Survey A and B with a subset of original respondents (468 and 303 respectively). All respondents aged 18 or above were recruited via a professional online panel. They were sampled using quotas for gender, age and place of residence (federal state). In Survey A (B) the average age was 47 (46) years; 53% (49%) of our respondents were male; 34% (34%) of our respondents have a higher education entrance certificate.

The surveys were structured as follows: First, all respondents were asked about the current level of their SRM awareness. Then they were all shown the same information video about SRM. The video provided respondents with information on SRM using animated graphics. The animations were supported by verbal explanations spoken by a professional radio presenter. Respondents who were not able to listen to or to play the video were excluded at the beginning of the survey. It was not possible to fast-forward the video or skip parts of it. The video first provided respondents with information on anthropogenic climate change and its likely consequences and explained the two-degree target. The video then introduced mitigation, adaptation and SRM as three possibilities of tackling climate change. Subsequently, the video explained SRM in more detail, i.e. its underlying mechanisms and its impact on climate change, the current state of research and the potential benefits and risks of SRM. The information was based on peer-reviewed papers and scientific reports (taken from, e.g., [47, 60–62]). External experts checked the information for correctness and clarity. An English translation of the German video script is provided in the Supporting Information (S2 File). After watching the video, we asked respondents about the clarity of the information provided on the video. For each survey, more than 98% of the respondents indicated that they had understood the video well or very well.

After this initial information stage on the subject of SRM, we proceeded to elicit the respondents’ acceptance of SRM. Respondents were asked about their level of agreement (or otherwise) ‘to using SRM to counteract climate change‘. Potential answers ranged from 1 (‘strongly disagree’) to 4 (‘strongly agree’). Next, respondents were asked to assess the risks and benefits of SRM using a scale from 1 (‘very small’) to 4 (‘very large). Subsequently, we elicited respondents’ WTP, using either the Point-WTP method (Survey A) or the Range-WTP method (Survey B). The WTP questions are described in more detail in section 3.2. We then elicited respondents’ emotional response to SRM. Respondents were asked how strongly they experienced various positive reactions (delight, satisfaction, hopefulness, relief) and negative responses (worry, fear, sadness, anger, annoyance) when thinking about SRM, using a scale from 1 (‘not at all’) to 4 (‘strongly’). In our analysis, we summarised negative and positive emotions in a composite index. Finally, we collected information on the respondents’ gender, age, income and education. A respondent with a higher-education entrance certificate is coded as having a high level of education.

Tables A-I in S1 Table contain summary statistics for all survey items used in our analysis.

### Willingness to Pay Elicitation

In both surveys, respondents were first asked to consider a hypothetical situation in which there were two potential ways of achieving the two-degree target, one being SRM, the other increased investment in renewable energy and improvement of energy efficiency (EE). Respondents were then asked whether they would prefer SRM or EE as a way of achieving the two-degree target. ‘Don’t know’ answers were also possible. Respondents thus first expressed their preferences for or against using SRM. We included this first question to ensure that respondents would state their WTP and not their WTA in the actual WTP question. In a follow-up question, respondents were then asked to assume that their chosen option would be more costly to realise than the alternative option. If, for instance, a respondent chose EE, we asked her to assume that it would be more costly to reach the two-degree target via EE than via SRM. Respondents were then asked to state their WTP either for the avoidance of SRM via EE (if they chose EE) or for the avoidance of EE via SRM (if they chose SRM). For the exact wording of the questions, see S1 File in the Supporting Information.

In Survey A we elicit Point-WTP. Taking the example of a respondent who chose EE over SRM, we asked her to state the maximum amount of money she would be willing to pay each month for the avoidance of SRM via EE. In Survey B, by contrast, we elicit Range-WTP. Respondents were asked to state a lower and an upper bound of WTP. Taking the same example of a respondent who chose EE over SRM, the lower bound of Range-WTP was elicited by asking the respondent to state the monthly amount of money that she would definitely be willing to pay to ensure avoidance of SRM via EE. The upper bound was elicited by asking the respondent to state the monthly payment *at*, *or above*, *which* she would definitely no longer prefer EE to SRM. We refer to the averages of the lower and upper WTP bounds as Range-WTP.

Regarding stated WTP values, our analysis considers only WTP values that are non-negative and smaller than EUR 500. Values above EUR 500 Euro were winsorised by setting them equal to EUR 500. In literature, winsorising is a standard approach to curtailing the influence of outliers (see e.g. [63]). Also, very high WTP values may simply reflect inaccurate answers or protest against the use of SRM. For Range-WTP we only consider WTP values if the stated value for the upper WTP bound is above the lower bound value. Finally, for respondents stating only one WTP value in the Range-WTP survey, we used this value as lower and upper bound, as it seemed legitimate to assume that those 12 respondents were certain about their preferences. However, excluding these observations leaves our results as described below unchanged.

### Validity and Reliability

We also compared and analysed the performance of the range and point methods with respect to theoretical validity and test-retest reliability. To address theoretical validity, we analyse whether the stated WTP values coincide and correlate with other objective or subjective factors. Logic suggests that these should be systematically related to the stated WTP values [64]. In this way, we not only elicited respondents’ WTP for the avoidance of SRM but also their self-reported acceptance of SRM, their risk perception for SRM and other perception measures. Obviously, these perception measures should be related to the respondents’ WTP. For instance, respondents who disagree with the use of SRM or who perceive the risks of SRM as severe should display a higher WTP value for the avoidance of SRM. We further analyse the test-retest reliability of the two methods by considering the correlation between the same respondents’ answers at two different points in time [57]. Furthermore, we test whether our results on theoretical validity are stable over time (section 4.2.4).

## Empirical Findings

We first describe our findings on acceptance and WTP for the two surveys separately (section 4.1), proceeding from there to compare the theoretical validity of the two WTP methods (section 4.2). We start by analysing how the correlation with acceptance differs between the two WTP methods (section 4.2.1). We then extend our analysis and also consider the correlation of WTP values with other perception measures such as risk perception (section 4.2.2). In addition, we report robustness checks, where we also show that all our results are robust to changes in the threshold of the WTP values (section 4.2.3). Finally, we test whether our results on theoretical validity are stable over time (section 4.2.4).

In the following, we restrict our analysis to the overwhelming majority of respondents in both surveys who chose EE (86% in Survey A, 90% in Survey B). We refer to the averages of the lower and upper WTP bounds as Range-WTP.

### Descriptive Findings

**Fig 2:**
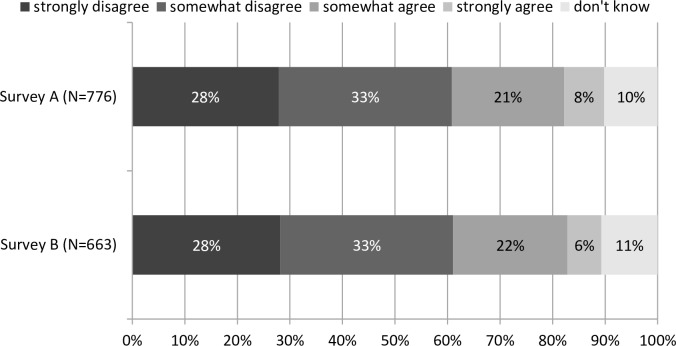
Acceptance of SRM in Survey A (Point-WTP) and Survey B (Range-WTP). Note: The survey asked the following question: ‘Please state your level of (dis)agreement with the following statement: We should use SRM to counteract climate change.’

Fig 2 illustrates our findings on the acceptance of SRM in the two surveys. In Survey A, 61% of all respondents stated that they disagreed either strongly or to some extent with the use of SRM to counteract climate change. By contrast, only 29% of the respondents agreed either somewhat or strongly with the use of SRM.

The figures are almost identical for Survey B. Here, 61% of the respondents disagreed with the use of SRM and only a minority of 28% agreed. Overall, we find virtually identical levels of acceptance in the two surveys (Wilcoxon rank sum test p = 0.6930). Moreover, the overwhelming majority in both surveys chose EE (86% in Survey A, 90% in Survey B) over SRM.

Up to this point, both surveys were exactly identical. In fact, we find almost identical levels of choice and acceptance of SRM in the two surveys. The results of the two surveys are, therefore, highly consistent. However, when we apply the two methods to eliciting the WTP of respondents and compare the WTP figures for both methods, we find major differences. For Survey A (Point-WTP) we find a mean WTP of EUR 44 for EE and a median WTP of EUR 20. Overall, 12% of the respondents reported a Point-WTP of zero. In Survey B (Range-WTP), we find that the mean of the lower bound WTP for EE is EUR 52 and the mean of the upper bound WTP value is EUR 126. The mean WTP value of the Range-WTP, the averages of the lower and upper WTP bounds, is EUR 89 (with a median of EUR 55). Here, 6% of the respondents reported a Range-WTP of zero.

Overall, we thus find that the stated WTP values differ greatly between Survey A and Survey B although the levels of acceptance do not. It is at first sight somewhat puzzling that the lower bound of Range-WTP exceeds Point-WTP. Wang et al. [35] argue that the lower bound should be close to Point-WTP and for chocolate their lower bound is also larger than Point-WTP. In our study, differences may also be due to the time interval between both surveys, probably due to an increasing awareness of climate change. In fact, in the follow-up studies, which we report on in section 4.2.4, Point-WTP and lower bound are nearly identical. A potential explanation for why the lower bound is close to the Point-WTP is that respondents take the lower bound as a reference point for their valuations. The lower bound therefore acts as an anchor for determining the Point-WTP. There is ample evidence that there is insufficient adjustment in the case of anchoring [65]. This implies in our case that the Point-WTP is close to the lower bound. However, in the present study we are not primarily interested in the absolute size of WTP measures but in their theoretical validity, which is what we analyse next.

### Theoretical Validity of Willingness to Pay Measures

#### Acceptance and Willingness to Pay

In a first step, we test whether the general decision to choose EE instead of SRM is negatively correlated with the acceptance of SRM. We expect a negative correlation: respondents who choose EE should also disagree with the use of SRM.

We find for both surveys that the choice of EE over SRM is strongly negatively correlated with the stated acceptance of SRM. The correlation is -0.4987 (p-value of 0.0000) in Survey A and 0.4766 (p-value of 0.0000) in Survey B. Therefore, as expected, respondents who disagree with the use of SRM also choose EE over SRM. This is true for both surveys, so the results of the surveys are again highly consistent.

In a second step, we then tested whether respondents who disagree (agree) strongly with using SRM to counteract climate change also revealed higher (lower) WTP for the avoidance of SRM. In the presence of theoretical validity, WTP for the avoidance of SRM should be negatively related with the acceptance of SRM. Surprisingly, we find that Point-WTP is not significantly correlated with acceptance. The correlation is low (0.0297) and insignificant, with a p-value of 0.4868. Using the Point-WTP method, we therefore find no evidence for the hypothesis that respondents who are less agreeable to using SRM also have higher WTP for the avoidance of SRM. Accordingly, the Point-WTP method does not seem to satisfy the theoretical validity criterion.

**Fig 3:**
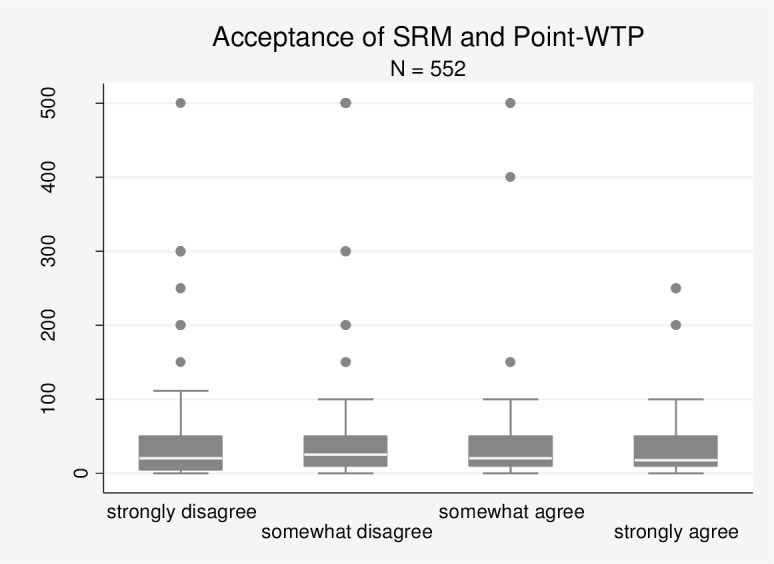
Boxplot diagram: Acceptance of SRM and Point-WTP (Survey A).

Further evidence comes from Fig 3, which visualises the relation between acceptance and Point-WTP in a boxplot diagram. Median WTP remains almost unchanged between the categories and is even the same for the categories of ‘strongly disagree’ and ‘somewhat agree’ (EUR 20). Theoretically, respondents who ‘strongly disagree’ with the use of SRM should state a higher WTP than respondents who ‘somewhat agree’ with its use. However, the boxplot diagram does not provide any evidence for this prediction. In fact, we find that median WTP is EUR 5 higher for those respondents who ‘somewhat disagree’ than for those who ‘strongly disagree’. This finding again runs counter to theoretical expectations.

In contrast to our previous result, we find a strong and statistically significant negative correlation of -0.2438 (p-value of 0.000) between acceptance and Range-WTP. We also find highly statistically significant correlations between acceptance and the upper bound (correlation of -0.2637, p-value of 0.0000) and lower bound (correlation of -0.1398, p-value of 0.0025) of Range-WTP. Accordingly, respondents who reveal lower levels of acceptance are indeed willing to pay more for the avoidance of SRM via EE. Using Range-WTP, we therefore find evidence for the theoretical proposition that acceptance of SRM and WTP for the avoidance of SRM are negatively related.

**Fig 4:**
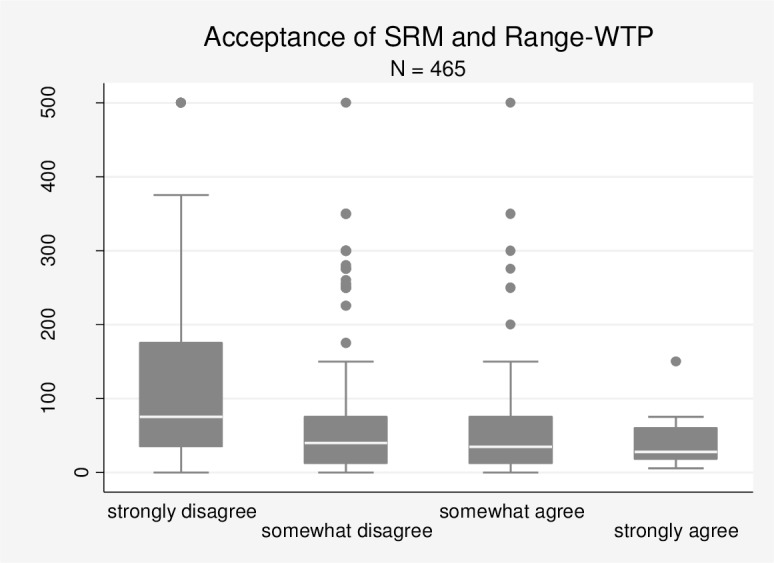
Boxplot diagram: Acceptance of SRM and Range-WTP (Survey B).

Fig 4 shows that the median of Range-WTP decreases with higher levels of acceptance. Median Range-WTP is EUR 75 for those who ‘strongly disagree’ but only EUR 27.5 for those who ‘strongly agree’. Therefore, the median declines by almost EUR 50 as we move from the lowest to the highest acceptance category.

We also control for systematic differences in respondents’ socio-demographic characteristics and their level of awareness of SRM by analysing the theoretical validity of both measures for subgroups of the sample.

Our findings on the correlation between acceptance and WTP also hold for different subgroups of the sample (defined by awareness, i.e. whether respondents had heard or not heard about SRM before the time of our survey, gender, education, age and income). Table 1 summarises these findings. For all different subgroups, not only Range-WTP but also its lower and upper bound are statistically significantly correlated with acceptance (results not shown). There is only one exception. The correlation between the lower bound and acceptance is not statistically significant for respondents aged 49 or above. In sharp contrast to the findings for Range-WTP, Point-WTP is not correlated with acceptance for any subgroup.

**Table 1:** Correlation of acceptance and WTP.

	Range-WTP	Point-WTP
All	-0.2438 ***	0.0297
	(0.0000)	(0.4868)
	N = 465	N = 552
Awareness: No	-0.2120 ***	0.0512
	(0.0001)	(0.2929)
	N = 355	N = 424
Awareness: Yes	-0.3216 ***	-0.0226
	(0.0006)	(0.8003)
	N = 110	N = 128
Men	-0.2454 ***	0.0110
	(0.0001)	(0.8465)
	N = 235	N = 312
Women	-0.2428 ***	0.0588
	(0.0002)	(0.3645)
	N = 230	N = 240
Education not high	-0.2154 ***	0.0748
	(0.0002)	(0.1627)
	N = 295	N = 350
Education high	-0.2954 ***	-0.0455
	(0.0001)	(0.5199)
	N = 170	N = 202
Age < = 49 years	-0.2955 ***	0.0613
	(0.0000)	(0.3242)
	N = 247	N = 261
Age >49 years	-0.1935 ***	-0.0048
	(0.0041)	(0.9347)
	N = 218	N = 291
Income < = 2500 Euro	-0.2170 ***	0.0792
	(0.0012)	(0.1786)
	N = 220	N = 290
Income > 2500 Euro	-0.2709 ***	-0.0377
	(0.0000)	(0.5431)
	N = 245	N = 262

Note: p-values in parentheses.

^*^ *p* < 0.10

^**^ *p* < 0.05

^***^ *p* < 0.01.

In summary, we find that Range-WTP is highly correlated with acceptance. In terms of theoretical validity, Range-WTP thus seems to outperform Point-WTP, which is not correlated with acceptance at all. Our analysis focuses on the large majority of respondents who chose EE. Yet, we find similar results when we focus on respondents who chose SRM instead. The Point-WTP is uncorrelated with acceptance (correlation coefficient 0.1239, p-value 0.2446, N = 90), whereas the Range-WTP is positively correlated with acceptance (correlation coefficient 0.3210, p-value 0.0230, N = 50).

#### Perception Measures and Willingness to Pay

We have shown that acceptance is not correlated with Point-WTP but highly negatively correlated with Range-WTP. We now analyse whether these findings also hold for other perception measures, such as risk and benefit perceptions or emotions about SRM. Our expectation is that respondents who perceive the risks of SRM to be more severe or who are more concerned about the use of SRM will also be willing to pay more for its avoidance. Table 2 shows how different perception measures are correlated with Range-WTP and Point-WTP.

**Table 2:** Correlation of perception measures and WTP.

Perception measures	Range-WTP	Point-WTP
Risk perception	0.2169 ***	-0.0304
	(0.0000)	(0.4749)
Benefit perception	-0.1458 ***	0.0184
	(0.0018)	(0.6677)
Negative emotions	0.2280 ***	-0.0521
	(0.0000)	(0.2134)
Positive emotions	-0.1516 ***	0.0539
	(0.0009)	(0.1998)

Note: P values in parentheses.

^*^ *p* < 0.10

^**^ *p* < 0.05

^***^ *p* < 0.01.

Again we find that, in terms of theoretical validity, Range-WTP performs much better than Point-WTP. Point-WTP is not correlated with any of the perception measures. By contrast, Range-WTP is significantly correlated with all perception measures. Range-WTP is, for instance, positively correlated with respondents’ risk perception. Respondents who perceive the risks of SRM to be appreciably severe are also willing to pay more for the avoidance of SRM. We also find a statistically significant positive correlation between Range-WTP and negative emotions towards SRM. Respondents who are concerned, angry or anxious display a higher WTP for the avoidance of SRM via EE. Also, the lower and upper bound of Range-WTP are statistically significantly correlated with all other perception measures (results not shown). There is only one exception. The correlation between the lower bound and benefit perception is not significant. Importantly, we find these relations only for Range-WTP and not for Point-WTP. The results strengthen our conclusion that the range method clearly outperforms the point method in terms of theoretical validity.

#### Robustness

As robustness check, we have set the threshold of EUR 500 to EUR 200 and winsorised the data by setting all WTP values of more than 200 to the upper bound of 200. Also, instead of winsorising the data to a certain threshold, we excluded all observations with WTP values of more than EUR 500 (200). Finally, we also excluded values above the 95% percentile. In all cases our results remain qualitatively unchanged. Details are available upon request.

#### Reliability and stability

In a follow-up surveys, we investigated whether our results were reliable and stable over time. First, we analysed the test-retest reliability of the two methods. To do so, we analysed the correlation between the same respondents’ WTP at two different points in time. Second, we checked whether our results on the theoretical validity of the WTP methods were stable over time. For these purposes, we repeated both surveys in August 2014 and again asked the respondents to state their acceptance and WTP. Overall, 468 of the original respondents participated in follow-up Survey A* (60%) and 303 in follow-up Survey B* (46%). As with the original surveys, both follow-up surveys were structured identically and differed only with respect to the WTP elicitation method. In follow-up Survey A*, respondents were again asked to state the Point-WTP, whereas in follow-up Survey B* respondents were again asked to state the Range-WTP. Note that the time difference between Survey A and its follow-up Survey A* is one year, whereas the time difference between Survey B and its follow-up Survey B* is one month. However, we do not expect the length of the time difference to influence the reliability and stability of our results. The time length should have a similar effect on both measures, WTP value and acceptance, so that the correlation between the two remains stable.

For both surveys, we find that WTP decreased over time. In follow-up Survey A*, the mean of the Point-WTP is EUR 32 (with a median of EUR 20) and is, therefore, almost EUR 12 lower than in the initial Survey A. In follow-up Survey B*, the mean of the Range-WTP is EUR 65 (with a median of EUR 35, a lower bound of EUR 33 and an upper bound of EUR 97) and is therefore EUR 24 lower than in the initial Survey B. Interestingly, the lower bound of the Range-WTP and the Point-WTP are now, as in the studies of Wang et al. [35], almost identical. The somewhat larger difference between the lower bound and the Point-WTP in the initial surveys may, therefore, indeed be due to the time interval between the initial Surveys A and B.

If we restrict the sample of the initial Surveys A and B to those respondents who participated in the follow-up survey, we also find a decrease in WTP. In fact, the mean Point-WTP for the restricted sample of the initial Survey A is EUR 43 and is, therefore, almost identical to the mean Point-WTP for the complete sample of the initial Survey A (mean Point-WTP of EUR 44). The mean Range-WTP for the restricted sample of the initial Survey B is EUR 87 (with a lower bound of EUR 48 and an upper bound of EUR 126) and is, therefore, also almost identical to the mean Range-WTP for the complete sample of the initial Survey B (mean Range-WTP EUR 89, with a lower bound of EUR 52 and an upper bound of EUR 126).

Analysing the test-retest reliability of the two methods, we find for both methods a high and statistically significant correlation between the WTP values over time (Point-WTP: correlation of 0.4469, p-value of 0.0000; Range-WTP: correlation of 0.4240, p-value of 0.0000). Therefore both methods fulfil the criterion of test-retest reliability.

Analysing the stability of our results over time, the two follow-up surveys confirm our previous results on the theoretical validity of the two WTP methods. For follow-up Survey A*, we find that the correlation between acceptance and Point-WTP is small (0.0095) and statistically insignificant (p-value of 0.8621). Accordingly, the Point-WTP again fails to satisfy the criterion of theoretical validity. For follow-up Survey B*, by contrast, we find that the correlation between acceptance and Range-WTP is strong (-0.2036) and highly statistically significant (p-value of 0.0042). If we restrict the initial samples of Survey A and Survey B to those respondents that participated in the follow-up survey, the above results are confirmed. We find no statistically significant correlation between acceptance and Point-WTP (p-value of 0.6949) but a highly statistically significant correlation for Range-WTP (p-value of 0.0002).

Table 3 shows how different perception measures are correlated with Range-WTP and Point-WTP for the follow-up surveys A* and B*. Again we find that, in terms of stability of theoretical validity over time, Range-WTP performs better than Point-WTP. Range-WTP is significantly correlated with all perception measures except for positive emotions. In contrast, Point-WTP is correlated with negative emotions, but is uncorrelated with risk and benefit perception and it even reveals the wrong sign for positive emotions.

**Table 3:** Correlation of perception measures and WTP.

Perception measures	Range-WTPSurvey A*	Point-WTPSurvey B*
Risk perception	0.2530*	0.0482
	(0.0005)	(0.3742)
Benefit perception	-0.1894 *	0.0890
	(0.0096)	(0.1050)
Negative emotions	0.2493 *	0.1054**
	(0.0003)	(0.0440)
Positive emotions	-0.0814	0.0873*
	(0.2422)	(0.0953)

Note: P values in parentheses.

^*^ *p* < 0.10

^**^ *p* < 0.05

^***^ *p* < 0.01.

Accordingly, Range-WTP also meets the criterion of theoretical validity in the follow-up survey, conducted a full year after the initial survey. This finding further strengthens our conclusion that the Range-WTP method clearly outperforms the Point-WTP method.

## Discussion and Conclusion

This paper compares two different open-ended methods of eliciting WTP. The first method, probably the most widely used method in practice, elicits WTP as a point. The second method elicits WTP as a range. One major criticism of the Point-WTP method is that it implicitly assumes that respondents know their preferences with certainty. Only under this assumption are they able to state one exact WTP value. By contrast, the Range-WTP method allows for preference uncertainty: respondents are free to state a range of values.

We compare the performance of both methods using the criterion of theoretical validity. The specific case we consider is WTP for the avoidance of SRM via increased investment in renewable energy and an improvement in energy efficiency (EE). At present, SRM is a relatively little-known technique for counteracting climate change and we expect respondents to exhibit a high degree of preference uncertainty. To analyse the theoretical validity of the two WTP methods, we conducted two large scale surveys in Germany eliciting respondents’ attitudes towards SRM and their WTP for the avoidance of SRM. The surveys were structured identically and differed only with respect to the respective WTP method. Survey A elicited WTP as a point, Survey B as a range. In two follow-up studies we repeated Survey A and B with the same set of respondents. We analysed the test-retest reliability of the two methods and studied whether the stated WTP values correlate over time. Furthermore, we analysed whether our results on the theoretical validity of the WTP methods were stable over time.

Our results are as follows: First, the stated WTP for the avoidance of SRM via EE differs strongly between the two surveys and thus depends on the WTP method used. Mean WTP elicited by the Point-WTP method is EUR 44, whereas mean Range-WTP is EUR 89. In contrast to Dost and Wilken [33] we thus cannot find any evidence that the mean of the Range-WTP equals the Point-WTP. The main reason for this difference seems to be the fact that Dost and Wilken [33] elicit valuations for a cup of coffee where, in sharp contrast to SRM, no preference uncertainties should be involved and market prices are known, i.e. where application of the range method seems limited. Nor did we find any evidence for the hypothesis mooted by Hakansson [41], who argues that the lower and upper bounds may provide a kind of confidence interval for Point-WTP. Our follow-up surveys confirm notably the results by Wang et al. [35], who find that the lower bound of Range-WTP is close to Point-WTP.

Second, we find a strong and statistically significant negative correlation between acceptance of SRM and Range-WTP. Respondents who disagree (agree) strongly with the use of SRM to counteract climate change also display a high (low) WTP for the avoidance of SRM. Accordingly, Range-WTP fulfils the criterion of theoretical validity. By contrast, acceptance and Point-WTP are uncorrelated. Therefore, the Point-WTP method does not meet the criterion of theoretical validity. Third, comparing the original with the follow-up surveys, we find that both methods fulfil the test-retest reliability criterion, since WTP values are highly correlated over both elicitations. Fourth, we show that the statistically significant negative correlation between acceptance and Range-WTP remains stable over time.

SRM is a relatively new technique that has hitherto been mostly discussed by experts and is largely unknown to the broader public. Yet the traditional Point-WTP method requires that respondents know their preferences with certainty and that they are familiar with the good in question so as to ensure that the elicited WTP values are reliable and valid [12]. By contrast, the Range-WTP method allows for preference uncertainty as it elicits more information about respondents’ preferences. While uncertain respondents are free to state a range of values, respondents who are certain about their preferences can also state equal lower and upper bounds. Therefore the range provides more information on respondents’ preferences than a single point. In our application, the upper bound of the range shows the strongest correlation with acceptance.

Our results support earlier criticism of the traditional open-ended Point-WTP method. We put forward and test an alternative open-ended approach, the Range-WTP method, which addresses this criticism. Given the increasing evidence on distortions induced by the use of closed-ended methods (i.e. starting point, range, and yea-saying biases) we believe that our results provide strong reasons to revive the interest in open-ended methods. Besides the richer set of information on individual preferences provided by open-ended methods they also have advantages for studies devoted to more than one country [10, 41]. Preference uncertainty could be regarded as one major reason for the poor empirical performance of open-ended Point-WTP; Range-WTP, at least in our studies, improves measurement substantially. Given these results, an important route for future research is to compare the performance of open- and closed-ended elicitation of WTP intervals.

## Supporting Information

S1 File Willingness to pay questions. (PDF)

S2 File Information provided in the SRM video. (PDF)

S1 Table Table A-I: Summary Statistics. (PDF)
