# Supplementary material for: Validity of Willingness to Pay Measures under Preference Uncertainty
Source: PLoS One. 2016 Apr 20;11(4):e0154078. doi: 10.1371/journal.pone.0154078 (PMC4838267; doi:10.1371/journal.pone.0154078)
Supplement: S1 File — (PDF) [file pone.0154078.s001.pdf]

**Willingness to pay questions**

**Introductory question**

Please consider the following situation:

Assume that the two-degree target is to be met. The international community sees two options for achieving the two-degree target:

1. Use of solar radiation management (SRM)

2. Reinforced expansion of renewable energies and improvement of energy efficiency (EE)

Assume first that, independently of the actual costs, both options would be equally costly. Which option would you prefer for achieving the two-degree target?

- ◯ Solar radiation management (SRM)
- ◯ Reinforced expansion of renewable energies and improvement of energy efficiency (EE)
- ◯ Don’t know

**Survey A: Point-WTP question**

**(If the respondent chose EE in response to the introductory question)**

Assume now that *reinforced expansion of renewable energies and improvement of energy efficiency (EE)* costs more than *solar radiation management (SRM)* and that EE would involve monthly costs for you.

Please answer the question assuming that you would actually have to pay the amount each month in addition to your monthly ongoing expenses for accommodation, accessory charges, daily needs and further purchases.

What is the maximum amount of money you would be willing to pay each month in order to achieve the two-degree target via reinforced expansion of renewable energies and improvement of energy efficiency (EE) rather than the use of SRM?

Please state the maximum amount here:

EUR________________________________

**Survey A: Point-WTP question**

**(If the respondent chose SRM in response to the introductory question)**

Assume now that *solar radiation management (SRM)* costs more than *reinforced expansion of renewable energies and improvement of energy efficiency (EE)* and that the use of SRM would involve monthly costs for you.

Please answer the question assuming that you would actually have to pay the amount each month in addition to your monthly ongoing expenses for accommodation, accessory charges, daily needs and further purchases.

What is the maximum amount of money you would be willing to pay each month in order to achieve the two-degree target via the use of solar radiation management (SRM) rather than EE?

Please state the maximum amount here:

EUR________________________________

**Survey B: Range-WTP question: (if the respondent chose EE in response to the introductory question)**

Assume now that *reinforced expansion of renewable energies and improvement of energy efficiency (EE)* costs more than *solar radiation management (SRM)* and that EE would involve monthly costs for you.

Please answer the question assuming that you would actually have to pay the amount each month in addition to your ongoing monthly expenses for accommodation, accessory charges, daily needs and further purchases.

Up to which level of costs for yourself would you definitely choose EE?
EUR________________________________

At or above which level of costs for yourself would you definitely no longer choose EE but SRM?

EUR________________________________

**Survey B: Range-WTP question: (if the respondent chose SRM in response to the introductory question)**

Assume now that *solar radiation management (SRM)* costs more than *reinforced expansion of renewable energies and improvement of energy efficiency (EE)* and that the use of SRM would involve monthly costs for you.

Please answer the question assuming that you would actually have to pay the amount each month in addition to your ongoing monthly expenses for accommodation, accessory charges, daily needs and further purchases.

Up to which level of costs for yourself would you definitely choose SRM?
EUR________________________________

At or above which level of costs for yourself would you definitely no longer choose SRM but EE?

EUR________________________________
