# Supplementary material for: Validity of Willingness to Pay Measures under Preference Uncertainty
Source: PLoS One. 2016 Apr 20;11(4):e0154078. doi: 10.1371/journal.pone.0154078 (PMC4838267; doi:10.1371/journal.pone.0154078)
Supplement: S2 File — (PDF) [file pone.0154078.s002.pdf]

Information Provided in the SRM Video

Sunlight warms the Earth and its atmosphere. Greenhouse gases in the atmosphere (e.g. CO_2) ensure that some of this warmth remains close to the Earth's surface. This makes the Earth warm enough for humans, animals and plants to live on.

Since the start of industrialisation around 1850, people have emitted a very large amount of greenhouse gases by burning coal, oil and gas. These gases trap more heat in the atmosphere and cause a gradual increase in average global temperature.

Since 1900, global temperature has risen by approximately 0.8°C. Almost all countries agree that the increase in average global temperature should not exceed two degrees compared to pre-industrial levels. The attempt to ensure that this is the case is referred to as the two-degree target.

By 2100, a further increase in temperature between 0.9 and 5.4°C is expected. The development depends strongly on the amount of greenhouse gases emitted in the future. To achieve the two-degree target, the current level of emissions would have to be cut by more than half by 2050. By 2100, greenhouse gas emissions would have to be reduced to almost zero.

It is virtually certain that climate change will cause a rise in sea levels. The frequency of heat waves is very likely to increase as well as the number of severe precipitation events in many regions. It is likely that in future more areas will be affected by extended droughts and that the frequency and intensity of tropical cyclones will increase. In addition, part of the CO2 emitted is absorbed by the ocean, thus causing ocean acidification.

There are different ways of dealing with climate change.

We can reduce greenhouse gas emissions or adapt to the new climate by building dikes. Another option is to reduce global temperature by means of solar radiation management (SRM).

Via SRM some sunlight is reflected before it can warm the Earth. This can be accomplished by, for example, spraying sulphate particles into the atmosphere at a high altitude.

A similar phenomenon can be observed in nature. When large volcanoes erupt, similar particles are distributed across wide areas of the Earth's atmosphere, cooling the Earth.

The particles remain in the higher regions of the atmosphere for approximately two years. To prevent the Earth from heating up again, spraying would have to be continued until the cause of global warming has been removed. Because CO2 remains in the atmosphere for a very long time, SRM might have to be used for several centuries. However, using SRM will not stop ocean acidification.

Currently, the risks, benefits and feasibility of SRM are the object of research.

The use of SRM entails benefits as well as risks. One of the benefits is that it would be a quicker way of counteracting global warming than cutting down on greenhouse gas emissions. This would buy additional time to remove the cause of climate change, i.e., the high concentration of greenhouse gases in the atmosphere. Massive and irreversible changes in the climate could be stopped before too much damage has been done. Furthermore, it would be possible to stop climate change even if certain countries decide not to reduce their greenhouse gas emissions. Deploying SRM would be less expensive than reducing the consumption of fossil fuels.

The risks include a change in the amount of precipitation in most regions. Arid regions in particular would have to cope with even less rain. If the deployment of SRM were suddenly stopped, global temperature would rise abruptly. The speed of this rise in temperature would lead to severe problems for humans and the environment. Because possible side effects would be trans-boundary, the use of SRM could cause international conflicts. Once in use, SRM might have a negative effect on people’s motivation to change their lifestyle: greenhouse gas emissions would continue to increase. Other hitherto unknown and unforeseeable risks might also arise.
