# Supplementary material for: Validity of Willingness to Pay Measures under Preference Uncertainty
Source: PLoS One. 2016 Apr 20;11(4):e0154078. doi: 10.1371/journal.pone.0154078 (PMC4838267; doi:10.1371/journal.pone.0154078)
Supplement: S1 Table — (PDF) [file pone.0154078.s003.pdf]

Table A-I: Summary Statistics

		Survey A		*Survey B*	
Variables	***Domain***	***mean***	***standard deviation***	***mean***	***standard deviation***
Awareness	0 (have never heard); 1 (have heard at least a little bit)	0.19	0.39	0.23	0.42
Acceptance	1 (strongly disagree) – 4 (strongly agree)	2.09	0.93	2.07	0.91
Risk perception	1 (very small) – 4 (very large)	3.23	0.74	3.29	0.74
Benefit perception	1 (very small) – 4 (very large)	2.45	0.78	2.43	0.77
Emotions (negative)*	1 (not at all) – 4 (strongly)	0.002	0.81	0.002	0.81
Emotions (positive)*	1 (not at all) – 4 (strongly)	-0.007	0.88	0.003	0.91
Gender	0 (male); 1 (female)	47%		51%	
High education	0 (other); 1 (A level)	34%		34%	
Income (monthly household income in €, net of taxes)	250 – 10,000	2400.47	1565.56	2483.25	1478.38
Age	18 – 87	47	15.42	46	15.21
N		776		663	

Note: * Mean values for emotions are averages of standardised items (mean = 0, standard deviation = 1). Items for negative emotions include worry, fear, sadness, anger, annoyance. Items for positive emotion encompass delight, satisfaction, hopefulness, relief.
